# Assessment of Patient, Physician, Caregiver, and Healthcare Provider-Related Factors Influencing “Glycemic Happiness” of Persons with Type 2 Diabetes Mellitus: An Observational Survey

**DOI:** 10.3390/clinpract11040087

**Published:** 2021-09-23

**Authors:** Sanjay Kalra, Vijaya Bhaskar Reddy Sagili, Debmalya Sanyal, Pradeep G. Talwalkar, Nareen Krishna Polavarapu, Kumar Gaurav, Amey Mane, Colette Stephen Pinto

**Affiliations:** 1 Bharti Hospital, Karnal 132001, India; brideknl@gmail.com; 2 Vijay Diabetes, Thyroid and Endocrine Clinic, Puducherry 600045, India; svbreddy1976@gmail.com; 3 Department of Endocrinology, KPC Medical College, Jadavpur, Kolkata 700001, India; drdebmalyasanyal@gmail.com; 4 Talwalkar Diabetes Clinic, Mumbai 400001, India; pg_talwalkar@hotmail.com; 5 Department of Medical Affairs, Dr. Reddy’s Laboratories Limited, Hyderabad 500016, India; Kumargaurav2@drreddys.com (K.G.); amey.mane@drreddys.com (A.M.); 6 Dr. Reddy’s Laboratories Limited, Mumbai 400001, India; colettestephenp@drreddys.com

**Keywords:** glycemic happiness, survey, type 2 diabetes mellitus, patients, physicians, caregivers, nurses, diabetes counselors

## Abstract

A multicentric cross-sectional observational survey was conducted to understand the patient, physician, nurse, caregiver, and diabetes counselor/educator-related factors that define the “glycemic happiness” of persons with type 2 diabetes mellitus (T2DM). Five sets of questionnaires based on a five-point Likert scale were used. A total of 167 persons with T2DM, 167 caregivers, and 34 each of physicians, nurses, and diabetes counselors/educators participated. For persons with T2DM, an adequate understanding of diabetes (mean score ± standard deviation: 4.2 ± 0.9), happiness and satisfaction with life (4.1 ± 0.8), flexibility (4.2 ± 0.8) and convenience (4.2 ± 0.7) of treatment, and confidence to handle hypo/hyperglycemic episodes (4.0 ± 0.9) were the factors positively associated with glycemic happiness. Caregivers’ factors included information from physicians on patient care (4.5 ± 0.6), constructive conversations with persons with T2DM (4.2 ± 0.8), helping them with regular glucose monitoring (4.2 ± 0.9), and caregivers’ life satisfaction (4.2 ± 0.8). Factors for physicians, nurses, and diabetes counselors/educators were belief in their ability to make a difference in the life of persons with T2DM (4.8 ± 0.4, 4.4 ± 0.5, and 4.5 ± 0.5), satisfaction from being able to help them (4.9 ± 0.3, 4.6 ± 0.5, and 4.6 ± 0.5), and professional satisfaction (4.9 ± 0.4, 4.4 ± 0.6, and 4.7 ± 0.4). Our survey identified the key factors pertaining to different stakeholders in diabetes care, which cumulatively define the glycemic happiness of persons with T2DM.

## 1. Introduction

There has been a steady increase in the burden of diabetes worldwide. According to the recent estimates from the International Diabetes Federation (IDF), 9.3% (463 million) of the total global population has diabetes, which is projected to reach 10.9% in 2045. Globally, India has the second-largest population of adults with diabetes (8.9%, with about 77 million people) [1]. The increasing burden of diabetes in India indicates the need for its optimized management to ensure better outcomes.

Diabetes is associated with complex social, behavioral, emotional, and environmental factors, which eventually affect the psychological “wellbeing” of persons living with diabetes and their therapeutic outcomes [2]. If left unattended, these psychosocial issues can negatively impact the wellbeing and social life of individuals with diabetes. Addressing such issues can help in overcoming the psychological barriers associated with adherence to treatment, self-care measures, and overall management of diabetes [3]. All the emotions related to day-to–day diabetes management and living with this chronic condition are broadly termed diabetes distress [4]. Kalra et al. defined diabetes distress as “an emotional response characterized by extreme apprehension, discomfort or dejection, due to perceived inability in coping with the challenges and demands of living with diabetes” [4]. According to the second Diabetes Attitudes, Wishes and Needs (DAWN2™) study for cross-national benchmarking of diabetes-related psychosocial outcomes in people with diabetes, 38.5% of the participants from India had diabetes distress, with India being ranked 11th among the other 17 countries in this category. Further, a majority of the participants reported diabetes to have a “slightly negative” to “very much negative” impact on their emotional wellbeing (47.5%) and relationship with family, friends, and peers (29.1%). Notably, only 31.5% of participants from India reported that their healthcare team enquired about anxiety or depression [5].

Several studies conducted across India have reported a high prevalence of diabetes distress, stress, and depression in people living with diabetes [6,7,8,9,10,11,12]. All this evidence clearly highlights the burden of diabetes distress, stress, and depression among people living with diabetes and the importance of life satisfaction and psychological wellbeing to ensure holistic management of diabetes in India.

It is well established that several stakeholders are involved in the multidisciplinary management of diabetes, including people living with diabetes, caregivers (friends and family), physicians, paramedical professionals such as nurses, and diabetes counselors [13]. Various factors associated with each of these stakeholders may influence (positively or negatively) and contribute to the “glycemic happiness” of people with diabetes. Glycemic happiness is defined as “a state of emotional and biomedical wellbeing in persons with diabetes mellitus” [14].

With the improved understanding of the impact of diabetes distress and the role of wellbeing on optimal outcomes in persons living with type 2 diabetes mellitus (T2DM), a few recent studies have evaluated the concept of “happiness” and its association with outcomes in these individuals [15,16,17]. There is, however, a dearth of literature to support an appropriate definition of glycemic happiness in people living with diabetes and identifying the key factors that contribute to an increase in happiness and subsequent optimal outcomes. With this background, an observational survey was designed to understand the patient, physician, caregiver, nurse, and diabetes counselor-related factors that can influence glycemic happiness or the overall emotional and biomedical wellbeing of persons with T2DM. The outcomes of the survey are discussed in the current article.

## 2. Methods

### 2.1. Study Setting and Participants

A multicentric cross-sectional observational survey was conducted between August 2020 and March 2021 in 34 centers across India. The survey involved persons with T2DM (aged ≥18 years) along with physicians, caregivers (family), diabetes nurses, and diabetes counselors/educators managing persons with T2DM. Individuals not willing to sign the informed consent form or participate in the survey were excluded.

### 2.2. Questionnaires Used for the Survey

The development of the questionnaires used in this survey has been described in detail in our previous report [14]. After a comprehensive review of several validated scales available for assessing the happiness and wellbeing of persons with T2DM and based on clinical experience, a group of expert diabetologists and endocrinologists from the country developed five sets of questionnaires to assess the parameters affecting the glycemic happiness of persons with T2DM, either directly or indirectly. Subsequently, the questionnaires were translated into multiple regional languages to facilitate easy data collection [14].

### 2.3. Data Collection

The survey was based on five sets of simple questionnaires developed by an expert panel and administered by designated personnel at study centers. Questions that would help correlate with glycemic happiness and help understand the factors that may influence the glycemic happiness of persons with T2DM either directly or indirectly were included in the survey forms. One questionnaire was used to record inputs from individuals with T2DM (patient component) while the remaining four questionnaires—physician component, caregiver component, nurse component, and counselor component—were answered by physicians, caregivers, nurses, and diabetes counselors/educators, respectively. The details of the questionnaires are included in Appendix A. All participants were asked to rate the parameters in their respective glycemic happiness survey questionnaires based on their degree of agreement regarding the relevance of the parameter for defining glycemic happiness on a Likert scale of 1 to 5, where 1 represented strongly disagree, 2 = disagree, 3 = neutral response (neither agree nor disagree), 4 = agree, and 5 = strongly agree. Vital signs of persons with T2DM were also recorded during the survey.

### 2.4. Ethics

This survey was conducted in accordance with the International Conference on Harmonization E6 Guideline for Good Clinical Practice, the Declaration of Helsinki (October 1996), and applicable local, state, and federal laws, as well as other applicable country laws (including the Indian Council of Medical Research regulations). Informed consent forms were obtained from all participants.

### 2.5. Statistics and Data Analysis

Data obtained were analyzed using R software. Continuous variables were presented as mean and standard deviation (SD), and categorical variables were presented as count and percentage. Mean scores and SD for each of the parameters were analyzed from the scoring given by each of the study participants. Parameters for which the mean scores were ≥4 were considered as key factors affecting the glycemic happiness of persons with T2DM.

## 3. Results

### 3.1. Demographic Characteristics of the Survey Participants

A total of 167 persons with T2DM, 167 caregivers, and 34 each of physicians, nurses, and diabetes counselors/educators managing persons with T2DM participated in this survey. All the participants completed the survey.

The demographic characteristics of persons with T2DM are provided in Table 1. The mean age of the participants was 55.4 years; the mean duration of diabetes was 106.6 months; the mean random blood glucose level was 183.7 mg/dL; and the mean glycated hemoglobin (HbA_1c_) was 8.1%.

The demographic characteristics of the caregivers, physicians, nurses, and diabetes counselors/educators are provided in Table 2.

### 3.2. Patient Component of the Survey

The outcomes of the patient component of the glycemic happiness survey have been enumerated in Table 3. Higher mean scores (in agreement with the questions) implying positive association with glycemic happiness were observed for the following parameters: satisfaction regarding their understanding of diabetes (mean ± SD: 4.2 ± 0.9), happiness and satisfaction with present life (4.1 ± 0.8), flexibility and convenience of their treatment (4.2 ± 0.8 and 4.2 ± 0.7, respectively), and their confidence in handling incidences of hyper- or hypoglycemia (4.0 ± 0.9).

### 3.3. Caregiver Component of the Survey

The responses from the caregiver component outcomes of the survey have been listed in Table 4. Among the parameters mentioned in the caregiver component of the questionnaire, high mean scores (in agreement) indicating the association with glycemic happiness of persons with T2DM were observed for the following parameters: receiving adequate information from doctors for providing care to persons with T2DM (4.5 ± 0.6), having constructive conversations with persons having T2DM to lower their anxiety (4.2 ± 0.8), being able to help persons with T2DM with their regular blood glucose monitoring (4.2 ± 0.9), and the caregiver’s happiness and life satisfaction (4.2 ± 0.8).

### 3.4. Physician, Nurse, and Diabetes Counselor/Educator Components of the Survey

The findings from the physician, nurse, and diabetes counselor/educator components of the survey have been listed in Table 5. For the physicians, nurses, and diabetes counselors/educators, high mean scores (for agreement) for a positive association with glycemic happiness of persons with T2DM were consistently observed for the following parameters: happiness and satisfaction for being a diabetes care professional (4.9 ± 0.4, 4.4 ± 0.6, and 4.7 ± 0.4); conviction in being able to make a difference in the life of persons with T2DM (4.8 ± 0.4, 4.4 ± 0.5, and 4.5 ± 0.5); and satisfaction from being able to help persons with T2DM (4.9 ± 0.3, 4.6 ± 0.5, and 4.6 ± 0.5). In addition, for the physician and diabetes counselor/educator components of the questionnaire, high mean scores showing a positive association with glycemic happiness were also noted for having confidence in managing complex problems of T2DM (4.5 ± 0.6 and 4.1 ± 0.7).

**Figure 1:**
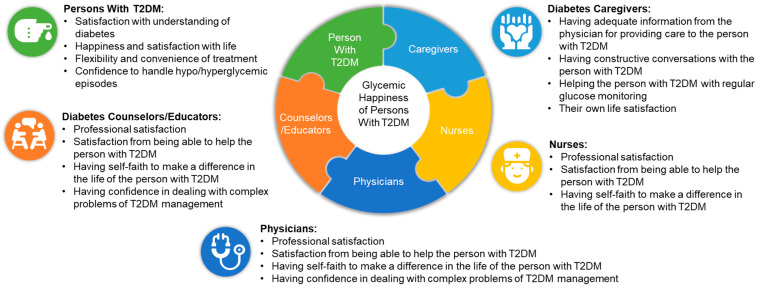
Patient, caregiver, nurse, physician, and diabetes counselor/educator-related factors affecting the glycemic happiness of persons with T2DM: Summary of findings from the survey. T2DM: Type 2 diabetes mellitus.

Figure 1 summarizes all the patient, caregiver, nurse, physician, and diabetes counselors/educator-related factors that affect the glycemic happiness of persons with T2DM, which are identified in this survey.

## 4. Discussion

To the best of our knowledge, this is the first of its kind survey to examine what constitutes glycemic happiness for persons with T2DM, by identifying emotional and biomedical wellbeing and defining parameters related to all the key stakeholders involved in diabetes care. The previous surveys involving persons with T2DM, physicians, and caregivers assessed the impact of various parameters, such as treatment satisfaction, life satisfaction, diabetes distress, depression, quality of life (QoL), and psychological wellbeing, on diabetes outcomes [18,19,20,21,22,23]. Although all these factors are related to the happiness of persons with T2DM, a comprehensive definition and assessment of the patient, physician, caregiver, nurse, and diabetes counselor/educator-related factors influencing the glycemic happiness of persons with T2DM were lacking. Here, we demonstrate that apart from factors concerning persons with T2DM, various parameters related to the people integrally involved in diabetes care can also cumulatively impact the glycemic happiness of persons with T2DM.

Several questionnaires and scales are in use for assessing parameters related to happiness among persons with T2DM. The diabetes distress scale assesses distress related to physicians, treatment distress, emotional burden, and interpersonal distress [6,7,10,24]. The GlucoCoper tool evaluates coping mechanisms in persons with T2DM, based on parameters such as acceptance, blame, planning, resistance, action, and optimism. It has been used as a screening tool to evaluate dysfunctional coping skills in pregnant women with diabetes [25]. The hypoglycemic confidence scale based on nine parameters evaluates the hypoglycemic confidence of persons with diabetes [26]. The diabetes treatment satisfaction questionnaire measures treatment satisfaction among persons with diabetes [20]. Other scales include the Problem Areas in Diabetes Scale for assessing emotional distress in persons with diabetes [27], Hypoglycemic Attitudes and Behavior Scale for measuring emotional aspects related to hypoglycemia, including avoidance, anxiety, and confidence [28]. Although these scales measure psychological and emotional aspects related to persons with diabetes, they do not directly assess their overall happiness. In this context, the present survey helps define various parameters which cumulatively describe the glycemic happiness of persons with T2DM. Importantly, the five-component questionnaire used in this survey captures not only parameters related to persons with diabetes but also those associated with caregivers, physicians, nurses, and counselors/educators who are actively involved in diabetes care.

The present survey was based on simple questionnaires with parameters that could be easily understood and interpreted, irrespective of medical background or training. Further, the scoring of the survey was also based on a simple five-point Likert scale, which was easy to interpret for any primary care physician or other healthcare providers. The interpretation of data was simple and based on the mean scores obtained from the total population corresponding to each set of questionnaires. Only those parameters with mean scores of 4 or higher were considered to be the key factors associated with glycemic happiness. Additional largescale studies across the country using these simple questionnaires might delineate a common landscape of parameters related to various diabetes care providers and patients, which cumulatively impact the glycemic happiness of persons with T2DM. Therefore, the importance of this study not only lies in the comprehensive identification of various factors affecting the glycemic happiness of persons with T2DM but also paves the way to facilitate effective materialization of personalized diabetes care in the country because glycemic happiness is increasingly being recognized as a critical determinant of diabetes outcomes. Moving forward, validation studies assessing glycemic happiness of persons with T2DM are warranted.

Numerous studies using questionnaire-based surveys have reported that the overall diabetes distress levels among persons with diabetes vary from 18% to 41.1% in India, while in the female population alone, distress levels could be as high as 46.5% [6,7,10]. Approximately 35% of people with diabetes have high/very high stress levels, while depression has been observed in 38% to 56.8% of the diabetic population [8,9,12]. In addition, studies to estimate life satisfaction levels, QoL, and psychological wellbeing in people with diabetes have also been conducted across the country [29,30,31,32]. A moderate level of satisfaction was reported in 54.4% of the population with diabetes, and 65% were not sure whether they were happy [30]. Insulin users were found to be less happy than noninsulin users [29]. Regular physical activity was associated with psychological wellbeing [31]. Diet satisfaction and general health were significantly associated with QoL [32]. Our survey indicates that a flexible and convenient treatment regimen, understanding of diabetes as a whole, and current satisfaction with QoL are important determinants of glycemic happiness in persons living with diabetes. Further, the glycemic happiness of persons with diabetes also depends on their confidence in dealing with glycemic fluctuations.

Our survey also looked at physician and caregiver components that could influence the glycemic happiness of persons with T2DM. To summarize, the physicians’ conviction, satisfaction with their choice of being a diabetes care professional, and their ability to deal with complex lifestyle issues could help define the glycemic happiness of persons with T2DM. The impact of physicians’ wellbeing and job satisfaction on their patients, team members, and healthcare organizations is well known; poor wellbeing and job dissatisfaction of physicians have been associated with patient dissatisfaction [22]. In the present survey, nearly all the physicians agreed that the satisfaction in their role as a diabetes care professional could positively influence the glycemic happiness of persons with T2DM whom they treated.

We report similar findings in the nurse and diabetes counselor/educator components of our survey. The nurses and diabetes counselors/educators agreed that their professional satisfaction, happiness and satisfaction from being able to help persons with T2DM, and their conviction in being able to make a difference in the lives of their wards could be the key parameters influencing the glycemic happiness of persons with T2DM. A systematic review of the role of nurses in diabetes care reported that nurses have a well-established role in motivating persons with diabetes, and they find it important to make their patients feel hopeful and secure [33]. A pragmatic randomized controlled trial in persons with T2DM has shown that in addition to standard diabetes care, a coach or diabetes counselor-led motivational interview of persons with T2DM significantly decreases psychological distress, as compared to standard care alone [34].

The glycemic happiness of persons with T2DM is also likely to be influenced by caregivers. In the present survey, the caregiver-related factors identified for determining glycemic happiness of persons with T2DM were caregivers’ life satisfaction, having adequate information from doctors to provide care, having constructive conversations with persons with T2DM to ease their anxiety, and helping persons with T2DM with regular monitoring of blood glucose.

Previous studies from India have shown that a high caregiving burden among diabetes caregivers increases their risk of depression and poor wellbeing [23,35]. Anxiety disorders are noted not only in persons with diabetes but also in diabetes caregivers [23]. It has been frequently reported that caregivers tend to feel isolated, tired, and overwhelmed. In the case of caregivers who are employed, missing workdays, quitting a job, or early retirement are often observed. Since persons with diabetes may extensively depend on caregivers, the wellbeing of caregivers has a positive impact on them [23]. The importance of proactive and friendly communication of the care provider in the happiness of individuals with diabetes has also been advocated by Kalra et al. [36]. In line with these findings, the present survey revealed that caregivers also felt that their happiness and satisfaction with life positively influenced the glycemic happiness of persons with diabetes.

Although the effect of the type of therapeutic interventions on the happiness of individuals with diabetes is beyond the scope of the present survey, previous studies have shown that the happiness of persons with diabetes varies with the type of intervention. Generally, insulin users have lower happiness than noninsulin users [15,29]. Rather than the type of treatment, our survey revealed that treatment convenience and flexibility were positively associated with glycemic happiness of persons with diabetes. Saatci et al. demonstrated that treatment satisfaction is associated with the general wellbeing of a person with T2DM [37]. Other studies have shown that the happiness of persons with diabetes is associated with physical activity and mindfulness training, which were not explored in the present survey [15,16]. In addition, although the dietary intake and social behavior of persons with T2DM may also affect their glycemic happiness [15], these aspects were not covered in our survey.

Glycemic happiness of persons with T2DM was found to be associated with their confidence in handling hypo- and hyperglycemic episodes. This reiterates the importance of diabetes education in the satisfaction and happiness of patients, which has been highlighted by other studies as well [38,39].

## 5. Limitations

Our survey had a few limitations. The first and foremost limitation is the small sample size since it was a pilot study. Secondly, it was a noninterventional survey; hence, it did not assess the impact of different treatment modalities on the glycemic happiness of persons with T2DM. Further, some other parameters that have been associated with glycemic happiness, such as social behavior, physical activity, mindfulness training, and diet, have not been assessed in this survey. Thirdly, this was a qualitative survey where translation, validity, and reliability were not pre-assessed. Finally, the findings of this pilot survey provide only a basis for further assessments and cannot be generalized to the diabetic population in India.

## 6. Conclusions

Diabetes care should encompass the entire spectrum, including amelioration of physical symptoms, reduction of the risk of complications, and promotion of wellbeing. Recent literature has reiterated the importance of the happiness of persons with diabetes in improving their outcomes. Several studies have assessed different components affecting the happiness of persons with diabetes, including satisfaction and emotional and psychological wellbeing. However, a comprehensive definition of glycemic happiness by identifying various parameters related to persons with diabetes and their associated diabetes care providers is lacking. This first of its kind survey aimed at understanding and identifying factors influencing the glycemic happiness of persons with T2DM. Satisfaction with life and adequate knowledge and education on diabetes were the key factors among persons with T2DM and their caregivers that affected the glycemic happiness of persons with T2DM. Confidence regarding hypoglycemia and helping persons with diabetes with positive communication and regular blood glucose monitoring were additional patient- and caregiver-related factors, respectively. Thus, among physicians, nurses, and diabetes counselors/educators, their psychological wellbeing and professional and life satisfaction were identified as potential factors affecting glycemic happiness of persons with T2DM. In addition, the confidence of physicians and diabetes counselors/educators in tackling complex problems of diabetes also influenced glycemic happiness. Therefore, the survey revealed several patient, physician, caregiver, nurse, and diabetes counselor/educator-related factors that cumulatively influence the overall glycemic happiness of persons with T2DM. These findings are important pointers for clinicians as we embark on a journey to deliver holistic care for our population with diabetes.

## Figures and Tables

**Table 1:** Demographic characteristics of persons with T2DM (*N* = 167).

Characteristics	Value
Gender, n (%)	
Male	90 (53.9)
Female	77 (46.1)
Age, years, mean ± SD	55.4 ± 12.0
Height, cm, mean ± SD	161.3 ± 9.0
Weight, kg, mean ± SD	70.8 ± 11.8
SBP, mmHg, mean ± SD	131 ± 19.5
DBP, mmHg, mean ± SD	81.5 ± 7.5
RBG, mg/dL, mean ± SD (*N* = 152)	183.7 ± 63.7
HbA_1c_, %, mean ± SD (*N* = 143)	8.1 ± 1.9
Diabetes duration, months, mean ± SD	106.6 ± 86.8

DBP: Diastolic blood pressure; HbA_1c_: Glycated hemoglobin; RBG: Random blood glucose; SBP: Systolic blood pressure; SD: Standard deviation.

**Table 2:** Demographic characteristics of caregivers, physicians, nurses, and diabetes counselors/educators.

Participants	Males, n (%)	Females, n (%)	Age, Years, Mean ± SD	Experience Years, Mean ± SD
Caregivers (*N* = 167)	77 (46.1)	90 (53.9)	44.8 ± 13.3	NA
Physicians (*N* = 34)	29 (85.3)	5 (14.7)	47.2 ± 8.7	18.4 ± 7.2
Nurses (*N* = 34)	4 (11.8)	30 (88.2)	31.6 ± 8.5	6.7 ± 4.6
Diabetes counselors/educators (*N* = 34)	9 (26.5)	25 (73.5)	32.6 ± 8.3	6.4 ± 4.2

NA: Not applicable; SD: Standard deviation.

**Table 3:** Mean Likert scale scores from the patient component of the glycemic happiness survey (*N* = 167).

Parameters from the Patient Component of the “Glycemic Happiness Survey”	Mean Score	SD
How satisfied are you with your understanding of your diabetes?	4.2	0.9
Do you feel that friends or family don’t appreciate how difficult living with diabetes can be?	3.1	1.4
How happy and satisfied are you with your life presently?	4.1	0.8
How flexible have you been finding your treatment to be recently?	4.2	0.8
How convenient have you been finding your treatment to be recently?	4.2	0.7
How confident do you feel that you know what to do when your blood glucose level goes higher or lower than it should be?	4.0	0.9
Do you feel your private and social leisure activities are impaired due to diabetes?	2.9	1.2
Do you feel that diabetes is taking up too much of your mental and physical energy every day?	2.9	1.3
Do you get angry, scared, and/or depressed when you think about living with diabetes?	2.7	1.3
Do you feel overwhelmed by the demands of living with diabetes?	2.9	1.1

SD: Standard deviation.

**Table 4:** Mean Likert scale scores from the caregiver component of the glycemic happiness survey (*N* = 167).

Parameters from the “Caregiver” Component of the “Glycemic Happiness Survey”	Mean Score	SD
Do you get adequate information from your doctor for providing care to your relative who has T2DM?	4.5	0.6
As a caregiver, do you have constructive conversations with the person with T2DM, when he or she experiences anxiety?	4.2	0.8
Being a caregiver, do you help the person with T2DM in regular blood glucose monitoring?	4.2	0.9
Do you accompany the person with T2DM during exercise/sports/other physical activity?	3.5	1.0
Do you feel dealing with hypoglycemia is one of the biggest challenges you face when it comes to being a caregiver of a person with T2DM?	3.6	1.1
As a caregiver, do you feel your personal physical and mental health is getting affected?	2.7	1.2
Do you feel you have to give up vacations, hobbies, or other social activities being a caregiver?	2.5	1.2
Do you feel you can keep your energy levels up while caring for the person with T2DM?	3.6	1.0
Do you get time to relax while caring for the person with T2DM?	3.9	0.8
How happy and satisfied are you with your life presently?	4.2	0.8

SD: Standard deviation; T2DM: Type 2 diabetes mellitus.

**Table 5:** Mean scores of the physician, nurse, and diabetes counselor/educator components of the glycemic happiness survey.

Parameters	Physicians (*N* = 34)	Nurses (*N* = 34)	Diabetes Counselors/Educators (*N* = 34)
	Mean ± SD	Mean ± SD	Mean ± SD
Do you feel happy and satisfied that you chose to be a diabetes care professional?	4.9 ± 0.4	4.4 ± 0.6	4.7 ± 0.4
Do you get satisfaction from being able to help T2DM patients?	4.9 ± 0.3	4.6 ± 0.5	4.6 ± 0.5
Do you feel you can make a difference in life of T2DM patients through your work?	4.8 ± 0.4	4.4 ± 0.5	4.5 ± 0.5
Do you get physically and emotionally exhausted at work?	2.6 ± 1.1	2.4 ± 1.0	2.2 ± 1.0
Do you feel you are losing enthusiasm at work?	2.0 ± 1.0	1.9 ± 0.8	1.9 ± 0.7
Do you feel you are in control dealing with complex problems of T2DM management?	4.5 ± 0.6	3.6 ± 0.8	4.1 ± 0.7
Do you feel worn out by your job as a care provider?	2.4 ± 1.3	2.3 ± 1.0	2.2 ± 0.9
Do you feel overwhelmed because diabetic patient load seems endless?	2.8 ± 1.2	2.4 ± 1.2	2.5 ± 1.0
Do you feel depressed by the traumatic stress of T2DM patients whom you try to help?	2.5 ± 1.1	2.4 ± 1.1	2.3 ± 1.1
Do you feel less empathetic and connected with your colleagues and friends?	2.4 ± 1.2	2.3 ± 0.9	2.1 ± 0.9

SD: Standard deviation; T2DM: Type 2 diabetes mellitus.

## Data Availability

No additional data were generated in this survey.

## Appendix A. Five-Component Questionnaire of the “Glycemic Happiness” Scale

**I Glycemic Happiness Scale for Patient Component**
❖ How satisfied are you with your understanding of your diabetes?
❖ Do you feel that friends or family don’t appreciate how difficult living with diabetes can be?
❖ How happy and satisfied are you with your life presently?
❖ How flexible have you been finding your treatment to be recently?
❖ How convenient have you been finding your treatment to be recently?
❖ How confident do you feel that you know what to do when your blood glucose level goes higher or lower than it should be?
❖ Do you feel your private and social leisure activities are impaired due to diabetes?
❖ Do you feel that diabetes is taking up too much of your mental and physical energy every day?
❖ Do you get angry, scared, and/or depressed when you think about living with diabetes?
❖ Do you feel overwhelmed by the demands of living with diabetes?
**II Glycemic Happiness Scale for Physician Component**
❖ Do you feel happy and satisfied that you chose to be a diabetes care professional?
❖ Do you get satisfaction from being able to help T2DM patients?
❖ Do you feel you can make a difference in life of T2DM patients through your work?
❖ Do you get physically and emotionally exhausted at work?
❖ Do you feel you are losing enthusiasm at work?
❖ Do you feel you are in control dealing with complex problems of T2DM management?
❖ Do you feel worn out by your job as a care provider?
❖ Do you feel overwhelmed because diabetic patient load seems endless?
❖ Do you feel depressed by the traumatic stress of T2DM patients whom you try to help?
❖ Do you feel less empathetic and connected with your colleagues and friends?
**III Glycemic Happiness Scale for Caregiver Component**
❖ Do you get adequate information from your doctor for providing care to your relative who has T2DM?
❖ As a caregiver, do you have constructive conversations with the person with T2DM, when he or she experiences anxiety?
❖ Being a caregiver, do you help the person with T2DM in regular blood glucose monitoring?
❖ Do you accompany the person with T2DM during exercise/sports/other physical activities?
❖ Do you feel dealing with hypoglycemia is one of the biggest challenges you face when it comes to being a caregiver of a person with T2DM?
❖ As a caregiver, do you feel your personal physical and mental health is getting affected?
❖ Do you feel you have to give up vacations, hobbies, or other social activities being a caregiver?
❖ Do you feel you can keep your energy levels up while caring for the person with T2DM?
❖ Do you get time to relax while caring for the person with T2DM?
❖ How happy and satisfied are you with your life presently?
**IV Glycemic Happiness Scale for Nurse Component**
❖ Do you feel happy and satisfied that you chose to be a diabetes care professional?
❖ Do you get satisfaction from being able to help T2DM patients?
❖ Do you feel that you can make a difference in the life of T2DM patients through your work?
❖ Do you get physically and emotionally exhausted at work?
❖ Do you feel you are losing enthusiasm at work?
❖ Do you feel you are in control dealing with complex problems of T2DM management?
❖ Do you feel worn out by your job as a diabetic care provider?
❖ Do you feel overwhelmed because your case load seems endless?
❖ Do you feel depressed by the traumatic stress of those whom you try to help?
❖ Do you feel less empathetic and connected with your colleagues and friends?
**V Glycemic Happiness Scale for Counselor/Educator Component**
❖ Do you feel happy and satisfied that you chose to be a diabetes care professional?
❖ Do you get satisfaction from being able to help T2DM patients?
❖ Do you feel that you can make a difference in the life of T2DM patients through your work?
❖ Do you get physically and emotionally exhausted at work?
❖ Do you feel you are losing enthusiasm at work?
❖ Do you feel you are in control dealing with complex problems of T2DM management?
❖ Do you feel worn out by your job as a diabetic care provider?
❖ Do you feel overwhelmed because your case load seems endless?
❖ Do you feel depressed by the traumatic stress of those whom you try to help?
❖ Do you feel less empathetic and connected with your colleagues and friends?
T2DM: Type 2 diabetes mellitus.
